# Detection of total and PRRSV-specific antibodies in oral fluids collected with different rope types from PRRSV-vaccinated and experimentally infected pigs

**DOI:** 10.1186/1746-6148-10-134

**Published:** 2014-06-17

**Authors:** Inge Decorte, Wander Van Breedam, Yves Van der Stede, Hans J Nauwynck, Nick De Regge, Ann Brigitte Cay

**Affiliations:** 1 Operational Direction Viral Diseases, Enzootic and (re)emerging diseases, CODA-CERVA, Groeselenberg 99, 1180 Ukkel, Belgium; 2 Operational Direction Interactions and Surveillance, CVD-ERA, CODA-CERVA, Groeselenberg 99, 1180 Ukkel, Belgium; 3 Department of Virology, Parasitology and Immunology, Faculty of Veterinary Medicine, Ghent University, Salisburylaan 133, 9820 Merelbeke, Belgium

**Keywords:** PRRSV, Oral fluid, Antibody detection, Collection method

## Abstract

**Background:**

Oral fluid collected by means of ropes has the potential to replace serum for monitoring and surveillance of important swine pathogens. Until now, the most commonly used method to collect oral fluid is by hanging a cotton rope in a pen. However, concerns about the influence of rope material on subsequent immunological assays have been raised. In this study, we evaluated six different rope materials for the collection of oral fluid and the subsequent detection of total and PRRSV-specific antibodies of different isotypes in oral fluid collected from PRRSV-vaccinated and infected pigs.

**Results:**

An initial experiment showed that IgA is the predominant antibody isotype in porcine saliva. Moreover, it was found that synthetic ropes may yield higher amounts of IgA, whereas all rope types seemed to be equally suitable for IgG collection. Although IgA is the predominant antibody isotype in porcine oral fluid, the PRRSV-specific IgA-based IPMA and ELISA tests were clearly not ideal for sensitive detection of PRRSV-specific IgA antibodies. In contrast, PRRSV-specific IgG in oral fluids was readily detected in PRRSV-specific IgG-based IPMA and ELISA tests, indicating that IgG is a more reliable isotype for monitoring PRRSV-specific antibody immunity in vaccinated/infected animals via oral fluids with the currently available tests.

**Conclusions:**

Since PRRSV-specific IgG detection seems more reliable than PRRSV-specific IgA detection for monitoring PRRSV-specific antibody immunity via oral fluids, and since all rope types yield equal amounts of IgG, it seems that the currently used cotton ropes are an appropriate choice for sample collection in PRRSV monitoring.

## Background

In the past, studies monitoring humoral immunity to specific pathogens relied primarily on humoral responses via serum samples. More recently, diagnosis based on analysis of oral fluids is rapidly gaining interest in both human and veterinary medicine, as collection of oral fluids is simple, cheap and non-invasive. Oral fluid is a clear, slightly acidic mucoserous exocrine secretion composed of more than 99% water [1]. Like in other mucosal secretions, the immunoglobulin fraction found in oral fluids predominantly consists of antibodies of the immunoglobulin (Ig) A isotype [2]. Mucosal IgA antibodies are actively produced in and secreted from the plasma cells of local glandular tissue, but can also enter the secretions when plasma cells capable of homing to the mucosa are stimulated and release local IgA [3]. IgG and IgM, which are mainly found in serum, are also present in oral fluids, albeit in lower quantities than IgA, and enter the oral fluid by way of passive leakage via gingival crevicular epithelium, although some may be locally produced in the gingiva or salivary glands [4]. The presence of IgA, but also of IgG and IgM, makes oral fluid a useful biological specimen for immunological assays.

For several human viruses, detection of specific IgA, IgG and IgM in saliva has already been evaluated to monitor the levels of virus-specific antibody immunity. Virus-specific antibodies to measles virus, cytomegalovirus, Epstein-Barr virus, human immunodeficiency virus, Puumala hantavirus, dengue virus and hepatitis C virus [5-11] have been detected in saliva. As expected, the predominant isotype of the virus-specific antibodies in saliva was virus- and time-dependent. Over the last few years, several studies have also evaluated the use of oral fluids as samples for use in antibody-based veterinary diagnostics. For instance, antibodies against several swine viruses, including African swine fever virus, classical swine fever virus, porcine circovirus type 2 and porcine reproductive and respiratory syndrome virus (PRRSV), have been detected in oral fluid specimens of pigs [12-16]. However, relatively little information is available on the amounts of virus-specific IgA, IgG and IgM antibodies present in oral fluids of virus-infected pigs. Moreover, all of the above studies in pigs use the oral fluid collection procedure initially described by Prickett et al. [17], which involves the use of cotton ropes. It is however possible that rope material has an important impact on the amount of antibodies and on the predominant antibody isotypes in samples collected via this method. For instance, oral fluid specimens collected from pigs with cotton or hemp contained higher amounts of PRRSV-specific IgG compared to samples collected with nylon rope [18].

The collection of oral fluids – by means of ropes – has a distinct advantage over the collection of blood samples – via venepuncture – regarding animal welfare, as it is less intrusive and pigs are more willing to cooperate. Moreover, it can greatly simplify sample collection for monitoring and surveillance purposes in swine herds, as samples can be collected at pen level as well as farm level. Therefore, the objective of the present study was to investigate the influence of different rope materials on the amounts and isotypes of total and virus-specific antibodies recovered from oral fluid samples of PRRSV-vaccinated or -infected animals.

## Methods

### Animals and housing

Eight female 8-week-old Belgian Landrace piglets of approximately 20 kg, purchased from a commercial swine herd known to be free of PRRS virus (TaqMan NA and EU PRRSV Reagents, Life Technologies) and negative for PRRSV-specific antibodies (PRRS X3 Ab Test, Idexx), were housed individually on slatted floors at air-filtered level-2 biosecurity facilities (CODA-CERVA, Machelen). Each pig was randomly assigned to control or vaccination or infection groups. Water and food were provided ad libitum. Animal experiments were performed in accordance with EU and Belgian regulations on animal welfare in experimentation. The protocol was approved by the joined ethical committee of CODA-CERVA and the Scientific Institute of Public health Belgium (procedure agreement no. 120112–01).

### Vaccination and infection

After one week of acclimatization, two experiments were conducted: a vaccination experiment and an infection experiment.

For the vaccination experiment, an attenuated PRRSV vaccine (Porcilis PRRS, Intervet) was used. The vaccine virus was diluted in Diluvac Forte according to the manufacturer’s instructions and each vaccine dose contained at least 10^4^ 50% tissue culture infectious doses (TCID_50_) of PRRS vaccine virus strain DV. Three pigs were vaccinated intramuscularly with 2 mL vaccine/pig at 9 and 11 weeks of age. One pig was not vaccinated and served as a control animal.

For the infection experiment, a 5th passage on MARC-145 cells of the PRRSV prototype strain Lelystad virus was used. Lelystad virus was cultivated and titrated on MARC-145 cells as described before [19]. A virus stock containing 1 × 10^5^ TCID_50_/mL was aliquotted and stored at -80°C until use. Three nine-week-old pigs were inoculated with PRRSV Lelystad virus via aerosol inoculation (10^5^ TCID_50_/nostril). One pig was not inoculated and served as a control animal.

### Sample collection

During the week before the start of the experiments, the animals were trained to chew on six different rope materials – two natural fibred ropes (cotton and hemp) and four synthetic ropes (polyester and polyamide (water absorbing); polypropylene and polyethylene (water repellent)) – by positive reinforcement.

In the vaccination experiment, “stimulated” oral fluids (oral fluids collected by masticatory or gustatory stimulation such as chewing [20]) were collected 1 day before the first vaccination and 14 days after each vaccination with four different rope materials according to procedures described by Prickett et al. [17]. In brief, one rope (length 1 m; diameter 14 mm) was suspended in each pen and was left in place for 30 min, during which the animal could chew on it and moisten it with oral fluid. At each time-point, animals were presented with the cotton ropes first, followed by hemp, polyamide and polyester. To recover the oral fluid samples, ropes were manually wrung and the oral fluids were collected in 50 mL conical centrifuge tubes (BD Falcon). “Unstimulated” oral fluid (oral fluid collected without exogenous gustatory, masticatory, or mechanical stimulation [20]) was collected at the same time points by means of suction with a small suction catheter. Prior to the oral fluid collection, animals were deprived of food for 8 h to prevent cross-reaction of food products with antibodies. Blood samples were collected from each pig at -7 and 28 days post vaccination using venepuncture.

In the infection experiment, stimulated oral fluids were collected with two different rope types (cotton first, followed by polyester) at -1, 14 and 28 days post infection as described above. Blood samples were obtained at the same time points. The time points for sample collection were chosen based on literature describing that PRRSV specific IgM and IgA antibodies could be detected at 14 days p.i. and IgG antibodies at 28 days p.i. in BAL fluids of PRRSV inoculated pigs [21]. All oral fluid samples were immediately chilled on ice, centrifuged at 1147 × *g* (Jouan CR312) for 10 minutes to remove insoluble materials, and stored at -80°C until use. From the blood samples, serum was collected, incubated for 30 min at 56°C and kept at -80°C until use.

All samples were first collected from the negative control animals, followed by the vaccinated animals and then the infected animals, in order to avoid contamination of the samples as much as possible.

### Detection of total IgA, IgG and IgM in oral fluid samples

Total amounts of IgA, IgG and IgM antibodies present in oral fluid samples from vaccinated animals were measured using a commercial direct sandwich ELISA (IgA, IgG and IgM (pig) – ELISA, Celltrend GmbH), following the manufacturer’s recommendations. Oral fluid samples were diluted 1:500 (IgA), 1:250 (IgG) and 1:50 (IgM) in phosphate-buffered saline (PBS), respectively. A standard curve was generated by assaying 2-fold dilutions of a standard concentrate containing 1000 ng/mL IgA (x-axis: log, Ig concentration; y-axis: linear, absorbance) and oral fluid results that fell within the linear part of the reference curve were extrapolated to determine concentrations, taking the dilution factor of the oral fluid samples into account. When the absorbance was outside the standard curve a subsequent determination with changed sample dilutions was carried out. Western Blotting was used to confirm ELISA results. Briefly, oral fluid samples were diluted 1:2 in PBS (pH 7.2) and mixed with NuPAGE Sample Reducing Agent (Life Technologies). The samples were separated by electrophoresis in 12% Tris–HCl gels (Bio-Rad) and transferred to polyvinylidene difluoride (PVDF) membranes (Bio-Rad) via Western Blotting. Membranes were blocked overnight in 5% non-fat dry milk in PBS containing 0.05% Tween-20 at 4°C. Membranes were consecutively incubated with a 1:50,000 dilution of biotinylated goat anti-swine IgA, IgG or IgM antibodies (AbD Serotec; 1.5 h, room temperature) and a 1:2,000 dilution of horseradish peroxidase (HRP)-conjugated streptavidin (GE Healthcare; 1.5 h, room temperature). Subsequently, labeled proteins were visualised using the SuperSignal West Dura Chemiluminescent Substrate ECL kit (Thermo Scientific). The emitted chemiluminescent signal was detected with a VersaDoc MP 4000 image analyser (Bio-Rad). ImageJ software (NIH Image) was used to estimate the relative amounts of protein (relative to antibody concentrations in unstimulated oral fluid).

### Detection of PRRSV-specific antibodies with IgA and IgG isotype in serum samples and oral fluid samples

PRRSV-specific IgG antibodies in sera were detected using the PRRS X3 Ab Test (Idexx) according to the manufacturer’s instructions. S/P ratios were calculated, using the following formula: S/P = (OD of sample – OD of negative control)/(OD of positive control – OD of negative control). Samples with an S/P ratio above 0.4 were considered positive for PRRSV-specific IgG antibodies. In addition, PRRSV-specific IgG antibody titres in sera were determined using the immunoperoxidase monolayer assay (IPMA) technique described by Labarque et al. [21]. Briefly, Marc-145 cells were seeded in 96-well cell culture plates, inoculated with PRRSV Lelystad virus and incubated for 24 h at 37°C and 5% CO_2_. Subsequently, culture medium was removed, cells were washed with PBS and dried at 37°C for 1 h. The plates were kept at -80°C until use. Plates were thawed and cells were fixed in 4% paraformaldehyde for 10 min and then washed twice with PBS. A solution of 1% H_2_O_2_ in methanol was added to the cells for 5 min, and cells were subsequently washed twice with PBS. 4-fold dilution series (starting dilution 1:10) of the sera in phosphate-buffered saline supplemented with 1% Tween 80 (PBS-T) and 10% negative goat serum were then added to the infected Marc-145 cells and cells were incubated for 1 h at 37°C. Subsequently, serum dilutions were removed and cells were washed with PBS-T. Cells were then incubated with peroxidase-labeled goat anti-swine IgG antibodies (Dako; dilution 1:500 in PBS-T + 10% negative goat serum) at 37°C for 1 h. Finally, the plates were washed with PBS-T and 50 μL of a substrate solution of 5% 3-amino-9-ethyl-carbazole in 0.05 M Na-acetate buffer (pH 5) supplemented with 0.05% H_2_O_2_ was added to each well and incubated at room temperature for 20 min. The reaction was stopped by replacing the substrate solution with Na-acetate buffer and the staining was analyzed via light microscopy (Axiovert 25, Zeiss). Positive and negative control samples were included on each plate.

In oral fluid specimens, PRRSV-specific IgA and IgG antibodies were detected using IgA- and IgG-based IPMAs, immunofluorescence assays (IFA), a commercial IgG-based ELISA (Idexx PRRS OF) and a commercial PRRSV ELISA modified for the detection of PRRSV-specific IgA in swine oral fluid [12]. The IPMA used for detection of PRRSV-specific IgA and IgG in oral fluid samples was a modified version of the IPMA for serum samples described above. Two-fold serial dilutions (starting dilution 1:2) of oral fluid samples were made in PBS and added to the infected Marc-145 cells. After 1 h of incubation at 37°C, oral fluid dilutions were removed and cells were washed with PBS-T. Subsequently, cells were incubated with biotinylated goat- anti-swine IgA (AbD Serotec; 1:10 in PBS) or biotinylated goat- anti-swine IgG (AbD Serotec; 1:10 in PBS) for 1 h at 37°C. Following a washing step with PBS-T, cells were incubated with Streptavidine-HRP (GE Healthcare; 1:20 in PBS) for 1 h at 37°C. Visualization of antibody-labeled, PRRSV-infected cells was identical as described above. For IFA, Marc-145 cells were seeded on glass inserts in 24-well plates, infected with PRRSV Lelystad virus and incubated for 24 h at 37°C and 5% CO_2_. Cells were then fixed in 100% methanol for 10 min at -20°C, air dried, and stored at -80°C until use. Cells were thawed and incubated with 1:2 dilutions of the oral fluids in PBS for 2 h at 37°C. Subsequently, cells were washed and incubated with either goat anti-swine IgA (dilution 1:10) (AbD Serotec) or goat anti-swine IgG (dilution 1:20) (AbD Serotec) for 1 h at 37°C. Finally, cells were washed and incubated with anti-goat FITC (dilution 1:50 or 1:100 when previously incubated with goat anti-swine IgA or IgG respectively) for 1 h at 37°C. Cells were washed, embedded in a glycerine-PBS solution (0.9/0.1 v/v) containing 2.5% 1, 4-diazabicyclo-(2,2,2)-octane, mounted and analysed using a fluorescence microscope (Dialux 20, Leitz Wetzlar) for FITC-stained PRRSV positive cells.

Oral fluid samples were also tested for the presence of PRRSV-specific IgG antibodies using the PRRS OF kit (Idexx), according to the manufacturer’s instructions. The OD values were converted into S/P ratios, using the formula as described above. Samples with an S/P ratio above 0.4 were considered positive for PRRSV-specific IgG antibodies. PRRSV-specific IgA in oral fluid samples was measured with a modified PRRS X3 Ab Test (Idexx) as described by Kittawornrat et al. [12]. Reference standard samples (=oral fluid samples collected from 37 pens at 0, 10, 15, 20, 28, 35, 41, 49, 56, 75, and 91 days after intramuscularly vaccination of 5-week-old pigs (*n* = ~1,100) with 2 ml of a type 2 PRRS MLV vaccine, kindly donated by prof. Zimmerman, College of Veterinary Medicine,

Iowa State University) were included in this modified test to verify its correct performance. The raw plate data were converted into S/P ratios, using the formula as described above with the OD values of reference samples at day 0 and 35 as negative and positive controls, respectively.

### Statistical analyses

Differences in oral fluid volumes collected with different rope materials; differences between IgA, IgG and IgM concentrations collected with a specific rope; and differences between ropes for IgA, IgG and IgM collection were compared by analysis of variance (ANOVA) and a Bonferroni post hoc test was applied for multiple comparisons. Statistical analyses were performed using SPSS Statistics V21.0 (IBM) software and *p* values ≤ 0.05 were considered to be significant.

## Results

### Influence of rope material on oral fluid collection

During the acclimatization period, animals were stimulated to chew on six different rope materials by positive reinforcement. The polypropylene and polyethylene ropes did not retain sufficient amounts of oral fluid and were therefore excluded from the rest of the experiment. All other rope types absorbed and released adequate amounts of oral fluid to allow further analysis. Nevertheless, for one animal at 28 days post vaccination (V1), not enough material was obtained from the synthetic ropes to allow IPMA and ELISA analysis. Pigs did not show a clear preference for any of the rope types. Mean oral fluid volumes (μl ± *SEM*) of 850 (±86.6), 2633.3 (±961.4), 2400 (±862.1) and 1900 (±1053.5) were collected with cotton, hemp, polyamide and polyester ropes respectively at 14 dpi, compared to 1750 (±433.0), 2833.3 (±1013.7), 666.6 (±317.9) and 1200 (±907.3) at 28 dpi. No significant differences were found in these mean oral fluid volumes, either at 14 or at 28 days post vaccination. Unstimulated saliva was more difficult to obtain and only volumes of 112.2 ± 16.8 μL (mean ± *SEM*) were collected.

### Analysis of total IgA, IgG and IgM concentrations in oral fluid samples collected from PRRSV-vaccinated animals

**Figure 1:**
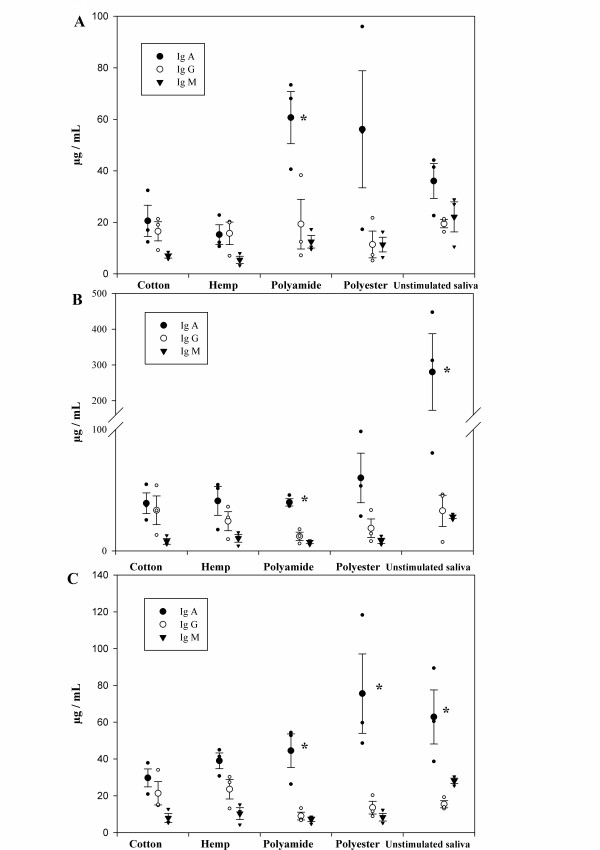
**Total IgA, IgG and IgM concentrations in porcine oral fluid samples as determined via ELISA.** Total IgA, IgG and IgM antibody concentrations in oral fluid samples collected directly or using different rope types at 0 **(A)**, 14 **(B)** and 28 **(C)** days post vaccination, were measured using a commercial direct sandwich ELISA (IgA, IgG and IgM (pig) – ELISA, Celltrend GmbH). Results represent the mean of three pigs. *Total IgA concentration is significantly different from total IgG and IgM concentration in this oral fluid sample (*p* < 0.1).

In order to determine whether rope material had an effect on the total amount of IgA, IgG and IgM recovered, total IgA, IgG and IgM concentrations were measured at three different time points during the vaccination experiment, using a commercial direct sandwich ELISA. The ELISA results (Figure 1) show that IgA is the predominant antibody isotype in unstimulated oral fluids (*p* < 0.05) at 28 days post vaccination (dpv), as well as in oral fluid samples collected using nylon ropes at 0, 14 and 28 dpv and polyester ropes at 28 dpv (*p* < 0.05). Interestingly, there were no significant differences between the IgA, IgG and IgM concentrations in samples collected using natural fibred ropes.

When comparing the different rope materials at a particular time point, no significant differences were found in the IgA/IgG/IgM concentrations, neither between samples collected with different ropes, nor between samples collected by means of ropes compared to unstimulated oral fluids. High individual differences between individual animals were however observed. Vaccination did not influence the total IgA, IgG or IgM concentrations present in oral fluids, nor the amounts collected by different ropes. Therefore, the results from the three different time points were also analyzed together, showing a significant difference between the rope types (P = 0.033). Two-by-two comparisons during the post hoc test showed that polyester ropes yielded significant higher amount of IgA than cotton (P = 0.011) and hemp ropes (P = 0.019) but equal amounts compared to polyamide ropes (P = 0.787).

**Figure 2:**
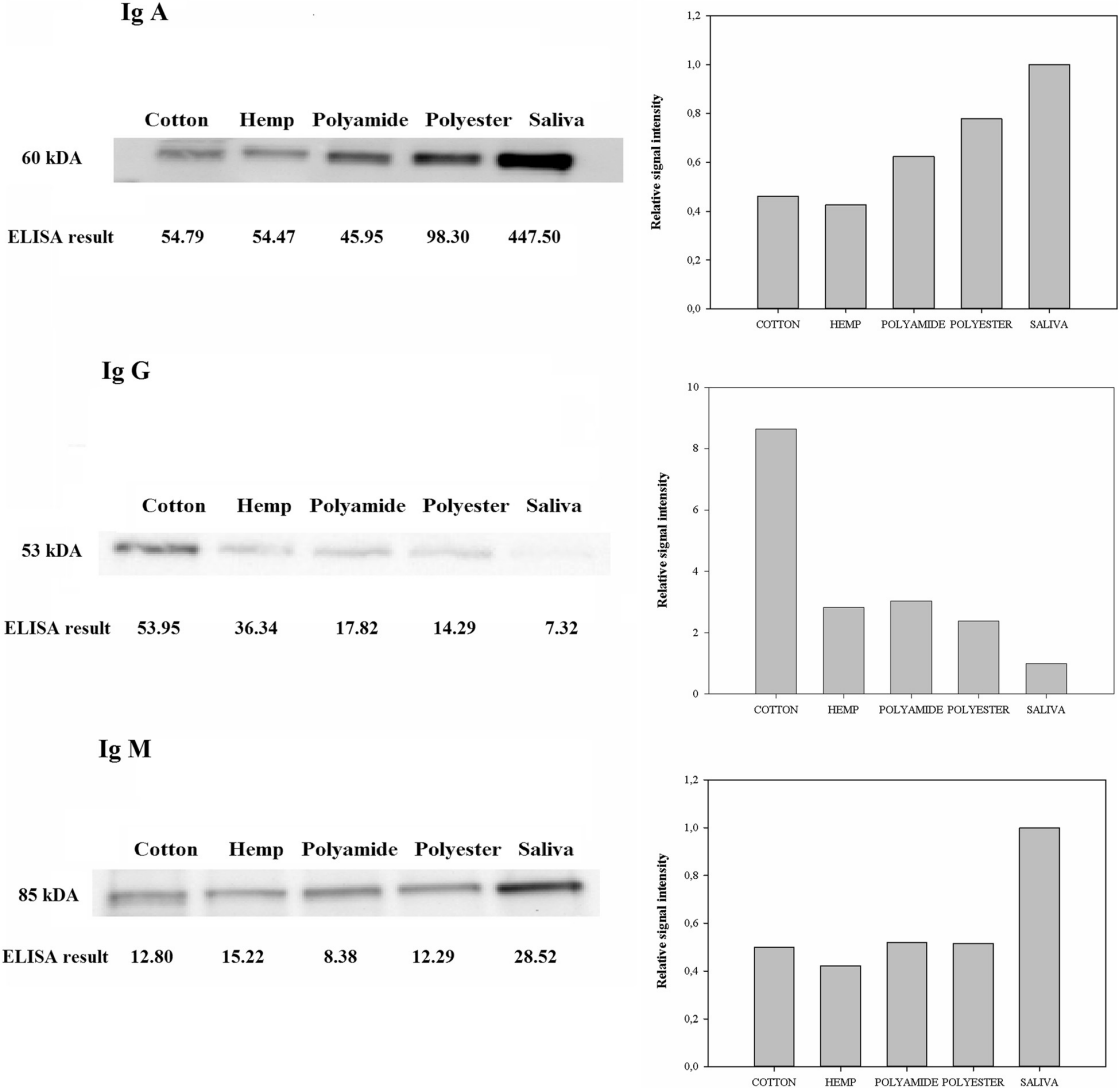
**Total IgA, IgG and IgM concentrations in porcine oral fluid samples as determined via Western Blot analysis.** Oral fluid samples were collected from one pig (V3) at 14 days post vaccination. The samples were reduced, subjected to SDS-PAGE and transferred to PVDF membranes via Western Blotting. Total IgA, IgG or IgM antibodies were detected using porcine IgA-, IgG- and IgM-specific antibodies. Specific bands of 60 kDa (IgA), 53 kDa (IgG) and 85 kDa (IgM) were detected. ImageJ software was used to quantify the relative amounts of protein and results were plotted relative to antibody concentrations found in unstimulated saliva.

To validate the results of the commercial direct sandwich ELISA, which has previously only been validated for serum samples, Western Blot assays were performed on oral fluid samples from one animal (V3), collected at 14 days post vaccination. Specific protein bands of the expected molecular weights (60 kDa, 53 kDa or 85 kDa) [22,23] were detected using swine IgA-specific, swine IgG-specific and swine IgM-specific antibodies, respectively (Figure 2). Quantification of the proteins using ImageJ software confirmed the results obtained with the direct sandwich ELISA.

### Detection of PRRSV-specific IgA and IgG antibodies in oral fluid and serum samples from PRRSV-vaccinated and experimentally infected animals

Immunoperoxidase monolayer assays (IPMA), immunofluorescence assays (IFA) and enzyme-linked immunosorbent assays (ELISA) were used to detect PRRSV-specific IgA and IgG antibodies in oral fluid samples collected with different rope types.

**Figure 3:**
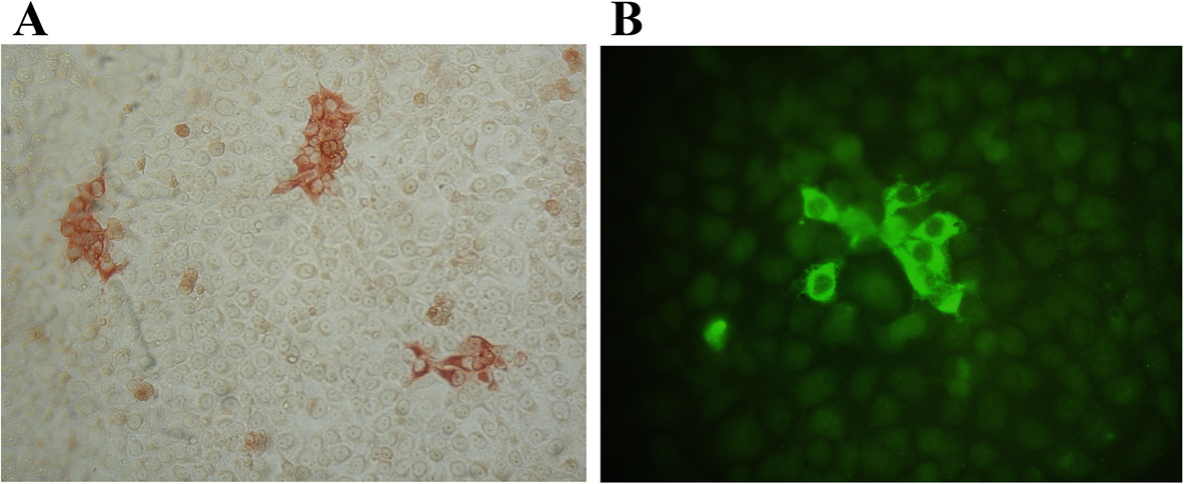
**PRRSV-specific IgG detection via immunoperoxidase monolayer assay (IPMA) and immunofluorescence assay (IFA).** PRRSV-infected cells were incubated with 1:2 dilutions of oral fluid samples. PRRSV-specific IgG antibodies bound to infected cells were subsequently detected via IPMA **(A)**, using biotinylated goat-anti-swine IgG antibodies and horseradish peroxidase-conjugated streptavidine, or IFA **(B)**, using goat-anti-swine IgG antibodies and FITC-conjugated anti-goat antibodies.

The PRRSV-specific IgA and IgG IPMA titers detected in oral fluid samples from PRRSV-vaccinated and experimentally infected pigs are shown in Table 1. For the vaccinated pigs, all oral fluid samples collected at 14 and 28 days post vaccination tested negative in IPMA for PRRSV-specific IgA antibodies. All oral fluid samples collected at 14 days post vaccination were also IPMA negative for PRRSV-specific IgG antibodies. However, at 28 days post vaccination, two out of three vaccinated animals showed positive IPMA titers of 2 and 4 for PRRSV-specific IgG antibodies when natural fibred ropes (cotton, hemp) were used for oral fluid collection (Table 1; Figure 3). Both these animals were also positive in IgG-based IPMA tests on serum samples collected at this time point. The third animal of the vaccinated group tested negative for PRRSV-specific IgG antibodies in oral fluid at 28 days post vaccination, but also tested negative in the IgG-based IPMA on serum collected at this time point. In the experimentally infected pigs, no PRRSV-specific IgA antibodies were detected via IPMA in any of the oral fluid samples. In contrast, PRRSV-specific IgG antibodies were readily detected at 14 and 28 days post infection in all oral fluid samples tested (collected with cotton or polyester ropes). The IPMA titers obtained with the oral fluid samples correlated with the IPMA titers obtained with the corresponding serum samples, but were markedly lower. The oral fluid IPMA results were confirmed by immunofluorescence staining: incubation of PRRSV-infected cells with oral fluids and subsequent detection with FITC-labeled goat anti-swine IgG antibodies resulted in immunofluorescent labeling of infected cells (representative immunofluorescence image in Figure 3) while detection with FITC-labeled goat anti-swine IgA antibodies did not result in any positive fluorescence signal (data not shown).

**Table 1:** PRRSV-specific IgA and IgG IPMA antibody titers of PRRSV-vaccinated and experimentally infected pigs

	**Post vaccination**												**Post infection**											
	**Ig A**						**Ig G**						**Ig A**						**Ig G**					
	**14 days**			**28 days**			**14 days**			**28 days**			**14 days**			**28 days**			**14 days**			**28 days**		
**Animal**	V1	V2	V3	V1	V2	V3	V1	V2	V3	V1	V2	V3	I1	I2	I3	I1	I2	I3	I1	I2	I3	I1	I2	I3
**Rope type**																								
*Cotton*	N	N	N	N	N	N	N	N	N	2	N	N	N	N	N	N	N	N	*	4	*	*	8	*
*Hemp*	N	N	N	N	N	N	N	N	N	4	2	N	‡	‡	‡	‡	‡	‡	‡	‡	‡	‡	‡	‡
*Polyamide*	N	N	N	*	N	N	N	N	N	*	N	N	‡	‡	‡	‡	‡	‡	‡	‡	‡	‡	‡	‡
*Polyester*	N	N	N	*	N	N	N	N	N	*	N	N	N	N	N	N	N	N	2	8	*	4	8	*
**Serum**	‡	‡	‡	†	†	†	‡	‡	‡	40960	10240	<10	†	†	†	†	†	†	640	2560	10240	160	163840	1024

PRRSV-specific IgA and IgG antibodies were respectively detected in sera and oral fluids using PRRSV-specific IgA- and IgG-based IPMA assays.

†: Tests not performed.

‡: Samples were not collected.

*Samples could not be tested due to insufficient material.

N: Negative.

When commercial ELISAs were used to detect PRRSV-specific IgG antibodies in oral fluid samples (PRRS OF, Idexx) and serum samples (PRRS X3 Ab Test, Idexx), no PRRSV-specific antibodies were detected at the start of the experiment (i.e. before vaccination/infection) (Table 2). In the vaccinated group, all oral fluid samples collected from all 3 vaccinated animals at 28 days post vaccination were positive in ELISA. However, only two out of the three vaccinated pigs had detectable serum antibody levels at this time point. In the PRRSV-infected group, all oral fluid and serum samples were ELISA positive at 14 days and 28 days post inoculation. When the modified ELISA protocol for the detection of PRRSV-specific IgA in swine oral fluid was used, S/P ratios for all oral fluid samples of the vaccinated group remained < 0.12 (Table 3). In the experimentally infected pigs, S/P ratios of 0.86 and 0.34 were found for oral fluid samples collected at 14 dpi with polyester ropes from pigs I1and I2, while all other samples had S/P ratios < 0.15. Unfortunately, oral fluid samples collected with cotton ropes could not be tested in the IgA-specific ELISA due to insufficient material.

**Table 2:** S/P ratio’s obtained by PRRSV-specific IgG ELISA tests for PRRSV-vaccinated and experimentally infected pigs

	**Post vaccination**									**Post infection**								
	**0 days**			**14 days**			**28 days**			**0 days**			**14 days**			**28 days**		
**Animal**	V1	V2	V3	V1	V2	V3	V1	V2	V3	I1	I2	I3	I1	I2	I3	I1	I2	I3
**Rope type**																		
*Cotton*	N	N	N	N	N	N	10.95	10.10	2.41	N	N	N	2.28	6.70	*	3.83	11.50	1.74
*Hemp*	N	N	N	N	N	N	11.64	10.86	2.61	‡	‡	‡	‡	‡	‡	‡	‡	‡
*Polyamide*	N	N	N	N	N	N	*	3.41	1.61	‡	‡	‡	‡	‡	‡			
*Polyester*	N	N	N	N	N	N	*	4.68	0.88	N	N	N	5.27	5.80	*	7.68	11.37	*
**Serum**	N	N	N	‡	‡	‡	2.15	1.32	N	N	N	N	0.88	1.09	2.24	1.46	1.91	0.77

PRRSV-specific IgG antibodies were detected in sera and oral fluids using the HerdChek* PRRS X3 ELISA kit (Idexx) and the PRRS OF kit (Idexx), respectively, according to the manufacturer’s instructions. For both assays, samples with an S/P ratio above 0.4 were considered positive for PRRSV-specific IgG antibodies.

‡: Samples were not collected.

*Samples could not be tested due to insufficient material.

N: Negative.

**Table 3:** S/P ratio’s obtained by a PRRSV-specific IgA ELISA test for PRRSV-vaccinated and experimentally infected pigs

	**Post vaccination**									**Post infection**								
	**0 days**			**14 days**			**28 days**			**0 days**			**14 days**			**28 days**		
**Animal**	V1	V2	V3	V1	V2	V3	V1	V2	V3	I1	I2	I3	I1	I2	I3	I1	I2	I3
**Rope type**																		
*Cotton*	-0.05	-0.00	-0.05	-0.03	-0.03	-0.02	0.11	-0.02	0.03	*	*	*	*	*	*	*	*	*
*Hemp*	-0.05	-0.03	-0.06	-0.01	-0.05	-0.04	0.07	0.01	0.04	*	*	*	*	*	*	*	*	*
*Polyamide*	-0.01	0.04	-0.02	0.03	-0.04	-0.01	*	-0.08	-0.00	*	*	*	*	*	*	*	*	*
*Polyester*	-0.08	0.09	-0.05	-0.05	-0.04	-0.04	*	-0.08	-0.01	*	*	*	0.86	0.34	*	0.03	0.14	*

PRRSV-specific IgA in oral fluid samples of PRRSV-vaccinated and experimentally infected pigs was measured with a modified PRRS X3 Ab Test (Idexx) as described by Kittawornrat et al. [12].

*Samples were not collected/available or could not be tested due to insufficient material.

Oral fluid samples and serum samples from the negative control pigs were found negative throughout the trial in IPMA, IFA and ELISA.

## Discussion

Oral fluid samples collected by means of ropes have the potential to replace serum samples for monitoring and surveillance of important swine pathogens [24]. So far, the most commonly used method to collect oral fluid samples is by placing a cotton rope in each pen and allowing the pigs to chew on the rope [17]. However, concerns about the influence of rope material on subsequent immunological assays have been raised [18]. In this study, the impact of rope material on total and virus-specific antibody detection in porcine oral fluid samples was assessed.

An initial experiment evaluated the oral fluid volumes that can be obtained using different rope types, and showed that waterrepellent ropes (polypropylene, polyethylene) retained insufficient oral fluid to perform downstream diagnostic assays. Consequently, waterrepellent ropes were excluded from the rest of the study. The amounts of oral fluid that could be collected with cotton, hemp, polyester and polyamide ropes were sufficient for downstream analysis and did not significantly differ from each other.

A recent study by Olsen et al. [18] showed that the rope material used for porcine oral fluid collection may affect downstream immunoassays to determine total IgM/IgA content [18]. Other recent studies assessed the effect of collection material on IgA retrieval from human saliva samples, and showed that cotton may interfere with immunoassay results by retaining water molecules, by releasing plant-hormones from the material or by binding of IgA to the cotton fibres [25,26]. Therefore, we determined the total amounts of IgA, IgG and IgM antibodies present in stimulated oral fluid samples collected with different ropes and in unstimulated porcine oral fluid samples. As expected, IgA was the dominant immunoglobulin fraction found in unstimulated oral fluids. Intriguingly, there were no significant differences in the IgA/IgG/IgM concentrations detected in unstimulated oral fluids and oral fluid samples collected using different rope types, indicating that no significant exclusion/retention of antibodies occurs in the rope materials.

No significant differences were observed between rope types when the three collection times were analyzed separately, indicating that all ropes are equally suitable for the collection of all three antibody isotypes. The observed high individual differences between pigs, together with the limited samples size, have however an important impact on the statistical analysis and make that future experiments using higher numbers of animals will have to be performed to confirm this conclusion. This is further underlined by the observation that when all samples of the three collection times are analyzed together, synthetic fibred ropes show to be more suitable for IgA collection than natural fibred ropes. Another aspect that has to be taken into account when interpreting these results is the fact that it cannot be excluded that the repeated sampling of the pigs with different rope types in a fixed collection order could have affected the results. In this aspect, Olsen et al. [18] already showed that in pen-based oral fluid sampling, the collection order does bias results in a non-uniform but statistically significant way.

**Figure 4:**
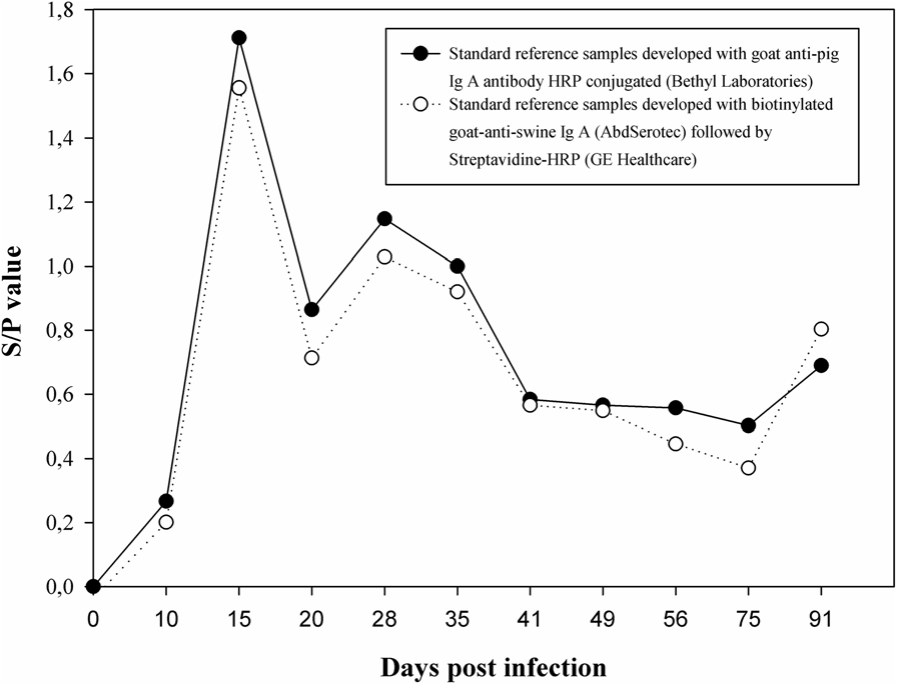
**ELISA detection of PRRSV-specific IgA antibodies in reference standard samples.** PRRSV-specific IgA in reference standard samples (kindly donated by prof. Zimmerman, College of Veterinary Medicine, Iowa State University) was measured with a modified PRRS X3 Ab Test (Idexx) as described by Kittawornrat et al. [12] that makes use of an HRP-conjugated goat-anti-pig IgA antibody (Bethyl Laboratories) PRRSV specific IgA in the reference samples was alternatively detected using the detection step of the IgA-specific IPMA, making use of biotinylated goat-anti-swine IgA antibodies (AbdSerotec) and Streptavidine-HRP.

Finally, we evaluated if IPMA and ELISA assays can be used for the sensitive detection of PRRSV-specific IgA/IgG antibodies in oral fluid samples of PRRSV-vaccinated or infected animals. In oral fluid samples of vaccinated pigs, no PRRSV-specific IgA antibodies were detected using either the IgA-specific IPMA or ELISA. This result was in line with the notion that intramuscular vaccination has a limited capacity to induce (protective) mucosal immunity [27]. In contrast, two oral fluid samples of experimentally infected pigs (collected at 14 dpi using polyester ropes) showed elevated S/P values (0.86 and 0.34) in the IgA-specific ELISA, suggestive for the presence of PRRSV-specific IgA. However, none of the oral fluid samples of infected pigs tested positive in the IgA-based IPMA. Interestingly, when a set of reference samples, including some with elevated S/P ratios in the IgA-specific ELISA (kindly provided by prof. Zimmerman), were tested in the IgA specific IPMA, all samples remained negative. These data suggest that the IgA-specific ELISA is more sensitive than the IgA-specific IPMA test. To exclude the possibility that the negative results in the IgA-based IPMA could be due to the use of non-functional porcine IgA-specific detection antibodies, the abovementioned reference samples were tested in a modified IgA-specific ELISA protocol using the detection step of the IgA-specific IPMA (biotinylated goat-anti-swine IgA followed by Streptavidine-HRP) instead of the standard detection step (HRP-conjugated goat-anti-pig IgA from Bethyl Laboratories). The modified and standard IgA-specific ELISA protocols yielded highly similar S/P values for the standard reference samples (Figure 4), indicating that the detection antibodies used for IgA-specific IPMA are functional and that the IgA-specific IPMA is indeed less sensitive than the IgA-specific ELISA. The presence of PRRSV-specific immunoglobulins of different isotypes in biological samples can (further) limit the sensitivity of (IgA-based) PRRSV-specific IPMA/ELISA assays, as antibodies with different isotypes may compete for the same/overlapping epitopes [28]. If it would be envisioned to develop or optimize IgA based diagnostic tests in the future, this could potentially be overcome by including an IgA capture step before evaluating the presence of PRRSV-specific antibodies.

Both the PRRSV-specific IgG-based ELISA and IPMA allowed sensitive detection of PRRSV-specific IgG antibodies in oral fluid samples. In vaccinated pigs, PRRSV-specific IgG was detected in oral fluid samples collected at 28 days post vaccination. In oral fluid samples of experimentally infected pigs, PRRSV-specific IgG was readily detected at both 14 and 28 dpi. When it is considered that IgG primarily enters the oral fluid by ways of passive leakage, these results are in line with the expected antibody response in serum upon vaccination or infection of pig with PRRSV respectively [29]. In general, there seemed to be a good correlation between qualitative results of the IgG IPMA in serum and oral fluid samples. Nevertheless, IgG IPMA titers were considerably lower in oral fluid samples than in serum samples. This discrepancy reflects the lower concentrations of IgG in oral fluid compared to blood: it was recently reported that the IgG concentrations in blood are approximately 800 times higher than in oral fluids [18]. Similarly as for the IgG IPMA data, a good correlation was observed between qualitative results obtained by IgG ELISA tests in oral fluid and serum samples.

Previous studies have reported that salivary IgG concentrations are less influenced by chewing to stimulate saliva flow or stress factors than IgA concentrations [30,31]. The results from our study indicate that the currently available tests for PRRSV-specific IgG detection in oral fluid samples are more sensitive than those for PRRSV-specific IgA detection in oral fluid, suggesting that IgG detection is preferable to IgA detection. The observation that the use of natural fibred ropes (cotton, hemp) may yield higher amounts of IgG suggests that the currently used cotton ropes are an appropriate choice for sample collection. Nevertheless, it should be noted that the inherent variability of natural fibred ropes can hamper standardization. The naturally wide variations in fiber quality, in combination with other factors that influence the fiber quality such as genetic variety, temperature, light intensity, herbivory by insects, irrigation method and nutrient stress [32], could potentially influence the diagnostic results.

## Conclusions

The results showed that IgA is the predominant antibody isotype in porcine oral fluid. Nevertheless, the PRRSV-specific IgA-based IPMA and ELISA tests were clearly not ideal for sensitive detection of PRRSV-specific IgA antibodies. In contrast, PRRSV-specific IgG in oral fluids was readily detected with PRRSV-specific IgG-based IPMA and ELISA tests, indicating that IgG is a more reliable isotype for monitoring PRRSV-specific antibody immunity in vaccinated/infected animals via oral fluids with the currently available tests. Since PRRSV-specific IgG detection seems more reliable than PRRSV-specific IgA detection for monitoring PRRSV-specific antibody immunity via oral fluids, and since the use of natural fibred ropes yields higher amounts of IgG, it seems that the currently used cotton ropes are an appropriate choice for sample collection in PRRSV monitoring.

## Abbreviations

Ig: Immunoglobulin; PRRSV: Porcine reproductive and respiratory syndrome virus; IPMA: Immunoperoxidase monolayer assay; IFA: Immunofluorescence assay; ELISA: Enzyme-linked immunosorbent assay; TCID_50_: 50% tissue culture infectious doses; HRP: Horseradish peroxidase; PBS: Phosphate-buffered saline; PBS-T: Phosphate-buffered saline supplemented with 1% Tween 80; ANOVA: Analysis of variance.

## Competing interests

The author(s) declare that they have no competing interests.

## Authors’ contributions

ID carried out the sample collection, optimised the diagnostic assays, carried out the statistical analysis and drafted the manuscript. YV participated in the draft of the manuscript and the statistical analysis. HN provided the virus and helped in drafting the manuscript. WVB assisted with the immunoassays and drafting of the manuscript. ND and BC conceived the study, participated in its design and coordination, and helped to draft the manuscript. All authors read and approved the final manuscript.

## Authors’ information

Nick De Regge and Ann Brigitte Cay share the last authorship.
